# Structural Insights into Substrate Binding and Antibiotic Inhibition of Enterobacterial Penicillin-Binding Protein 6

**DOI:** 10.3390/life12071022

**Published:** 2022-07-09

**Authors:** Mohd Zulkifli Salleh, Kirnpal Kaur Banga Singh, Zakuan Zainy Deris

**Affiliations:** Department of Medical Microbiology & Parasitology, School of Medical Sciences, Universiti Sains Malaysia Health Campus, Kubang Kerian 16150, Malaysia; m.z.salleh@usm.my

**Keywords:** *Shigella sonnei*, penicillin-binding protein 6, pentapeptide binding, antibiotic inhibition, homology modelling, molecular docking

## Abstract

*Shigella sonnei* remains the second most common cause of shigellosis in young children and is now increasingly dominant across developing countries. The global emergence of drug resistance has become a main burden in the treatment of *S. sonnei* infections and β-lactam antibiotics, such as pivmecillinam and ceftriaxone, are recommended to be used as second-line treatment. They work by inhibiting the biosynthesis of the peptidoglycan layer of bacterial cell walls, in which the final transpeptidation step is facilitated by penicillin-binding proteins (PBPs). In this study, using protein homology modelling, we modelled the structure of PBP6 from *S. sonnei* and comprehensively examined the molecular interactions between PBP6 and its pentapeptide substrate and two antibiotic inhibitors. The docked complex of *S. sonnei* PBP6 with pentapeptides showed that the substrate bound to the active site groove of the DD-carboxypeptidase domain, via hydrogen bonding interactions with the residues S79, V80, Q101, G144, D146 and R240, in close proximity to the catalytic nucleophile S36 for the nucleophilic attack. Two residues, R240 and T208, were found to be important in ligand recognition and binding, where they formed strong hydrogen bonds with the substrate and β-lactams, respectively. Our results provide valuable information on the molecular interactions essential for ligand recognition and catalysis by PBP6. Understanding these interactions will be helpful in the development of effective drugs to treat *S. sonnei* infections.

## 1. Introduction

*Shigella sonnei* is one of the four causative agents of shigellosis, an intestinal infection that is also known as bacillary dysentery. The species is considered as the second most common cause of diarrhea in young children in rapidly industrializing countries and together with *Shigella flexneri*, the species is responsible for more than 90% of the global shigellosis cases [1]. Although *S. sonnei* is traditionally isolated most commonly in developed countries, the species is now increasingly dominant and undergoing an unprecedented global spread across developing countries in Asia, Latin America, and the Middle East [2]. *S. sonnei* has an evolutionary advantage over *S. flexneri* and shows an extraordinary ability to acquire antimicrobial resistance genes, such as extended-spectrum beta-lactamase (ESBL) genes, from other pathogenic bacteria, most particularly *Klebsiella* spp. and *Escherichia coli* [3,4].

The global emergence of drug resistance, limiting the choice of effective antimicrobial drugs for shigellosis treatment, has become the main burden in the treatment of *S. sonnei* infections. In 2005, the WHO published guidelines for the treatment of shigellosis with a recommendation of ciprofloxacin as the first-line antimicrobial therapy for children. In cases of resistance to ciprofloxacin, pivmecillinam and ceftriaxone were recommended as the second-line treatment in all age groups, whereas azithromycin was recommended to be used exclusively in adults [5]. Antibiotics such as ampicillin, tetracyclines, chloramphenicol, nalidixic acid and trimethoprim/sulfamethoxazole were highlighted as inappropriate due to increasing antimicrobial resistance [5,6]. β-lactam antibiotics, such as pivmecillinam, an active prodrug of mecillinam (extended-spectrum penicillin) and ceftriaxone, a third-generation cephalosporin, work by inhibiting the biosynthesis of the peptidoglycan layer of bacterial cell walls, in which the final transpeptidation step in the peptidoglycan synthesis is facilitated by penicillin-binding proteins (PBPs), also known as DD-transpeptidases [7,8]. Mecillinam binds exclusively to PBP2 in Enterobacteriaceae and is usually used synergically in combination with other β-lactam antibiotics that bind to other PBPs, such as cefsulodin (PBP1s) and aztreonam (PBP3) [9]. Similarly, ceftriaxone mimics the D-alanyl-D-alanine moiety and binds to PBPs to inhibit the cross-linking of the peptidoglycan polymers. 

PBPs are classified into two subgroups, high-molecular mass (HMM) and low-molecular mass (LMM) [8,10]. Most bacteria possess several PBPs. For instance, *E. coli* possesses twelve PBPs, in which five of them are HMM PBPs and seven are LMM PBPs [8]. There are two primary D-Ala-D-Ala carboxypeptidases in *E. coli*, namely PBP5 and PBP6, which account for 85% of all PBPs in the organism [11,12]. PBP5 and PBP6 are LMM PBPs, which are involved in the cleavage of the peptide bond between two D-alanines of the peptidoglycan structure. All PBPs contain unique conserved motifs S-*X*-*X*-K, S-*X*-N and K-T-G, forming the active sites essential for the substrate catalysis [8,11]. The S-*X*-N motif, for example, is important in deacylation of the acyl–enzyme complex of the *E. coli* PBP5 and is modulated by its adjacent loop, formed by residues 74–90. Deletion of the loop, which has extensive contacts with the motif, completely abolished the carboxypeptidase activity [13]. The majority of D-Ala-D-Ala carboxypeptidases have been studied using PBP5 as a model system [13,14,15,16,17,18,19,20]. The crystal structures of PBP6 from *E. coli* have been determined previously with the antibiotic ampicillin and with a peptidoglycan substrate fragment containing the full pentapeptide [12]. The structures provide insights into the molecular interactions, vital for ligand binding and catalysis by D-Ala-D-Ala carboxypeptidases.

More importantly, a similar 43.8-kDa outer membrane-associated protein from *S. sonnei* has been shown to uniquely recognize immunoglobulin A (IgA) and IgG from patients previously infected by the bacterium, and interestingly, the protein did not cross-react with sera from patients that have been infected with other enteric infections [21]. Identification of such an antigenic protein that is specifically recognized by host antibodies would, therefore, guide us in the development of a new, fast and highly sensitive antigen-based test for specific detection of *S. sonnei* infections. In addition, it provides vital information on human immune responses against shigellosis. Albeit important, molecular characterization and structural details of the antigenic protein are scant. 

Our mass spectrometry analysis showed that the 43.8-kDa antigenic protein from *S. sonnei* is PBP6. In this study, we constructed the 3D-model of the 43.8-kDa antigenic protein from *S. sonnei* using protein homology modelling. We analyzed further the molecular complex of the protein with its substrate, peptidoglycan fragment containing the full pentapeptide, and expounded structurally in detail the molecular interactions between the protein and its potential antibiotic inhibitors, namely pivmecillinam and ceftriaxone, which are recommended by the WHO to be used to treat *S. sonnei* infections [6]. The results showed that the antigenic protein is a D-Ala-D-Ala carboxypeptidase and shares similar architecture particularly with PBPs 5/6 from *E. coli* [12]. These findings provide valuable information on the molecular interactions important for ligand recognition and catalysis by the protein. Moreover, our molecular docking revealed that the next-generation β-lactam antibiotics bind strongly to the active site of the protein. Understanding these interactions will be helpful to the development of effective drugs to treat *S. sonnei* infections.

## 2. Methodology

### 2.1. Protein Identification

The 43.8-kDa protein from *S. sonnei* has been shown to uniquely cross react with human IgA and IgG [21]. However, the 3D structure of this protein is yet to be determined. Our previous mass spectrometry analysis using matrix-assisted laser desorption/ionization-time of flight (MALDI-ToF) revealed that the 43.8-kDa protein from *S. sonnei* is PBP6. The protein sequence of the 43.8-kDa protein from *S. sonnei* (GenBank: CSE36004.1) was used and compared using BLAST (Basic Local Alignment Search Tool), available at https://blast.ncbi.nlm.nih.gov/Blast.cgi (accessed on 15 May 2022), against the non-redundant protein sequences database and multiple sequence alignment was carried out using Clustal Omega on Jalview [22] to examine sequence homology among the bacterial species. The results showed that the antigenic protein was a PBP6 with a D-Ala-D-Ala carboxypeptidase architecture and had a high degree of similarity with PBP6 from *E. coli* [12] and other *Shigella* species.

### 2.2. Protein Homology Modelling

BLAST search was performed again using the sequence of the 43.8-kDa protein from *S. sonnei* against the Protein Data Bank (PDB) database to search for suitable templates used in the protein homology modelling. A total of 9 PBP structures (PDB accession numbers: 3IT9 [12], 1NZO [13], 5J8X [15], 1HD8 [16], 6NTZ [14], 5TR7, 3A3J [17], 5FSR [23] and 4K91 [18]) were selected and used in the multiple sequence alignment prior to homology model construction using EasyModeller 4.0 [24]. The target sequence was aligned with templates and used to generate three models with the best discrete optimized potential energy (DOPE) scores. The best model was selected, optimized and subsequently used in the loop refinement tool, as implemented in EasyModeller 4.0 [24]. Verify3D [25] and PROCHECK [26] were used to verify the protein model with its 3D profiles and evaluate the stereochemical quality of the model by Ramachandran plot, respectively. Both programs were available at UCLA SAVES v6.0 (https://saves.mbi.ucla.edu/, accessed on 20 May 2022).

### 2.3. Protein and Ligand Molecular Docking

PBPs are key players in the peptidoglycan biosynthesis and remodeling of bacterial cell walls, as well as in drug resistance mechanisms [8,10]. As the name suggests, PBPs are the main targets of the β-lactam antibiotics and antibiotics such as pivmecillinam and ceftriaxone were recommended by the WHO for the treatment of shigellosis [6]. In order to understand the mechanism of inhibition exhibited by these β-lactam antibiotics, detailed intermolecular interactions between the antibiotic inhibitors and PBP6 from *S. sonnei* are crucial. Using the structures of pivmecillinam and ceftriaxone, retrieved from PubChem and converted into mol2 files, protein-ligand docking analysis was performed using AutoDock Vina, as implemented in UCSF Chimera [27]. Molecular docking simulations were executed at the catalytic active sites S36, K39, S102, N104, K205 and G207, by adopting the docking grid size of 28 × 30 × 28 Å along three axes, covering all the essential passive residues centered at the 9.24, −23.08, −1.27 Å regions, to provide enough space for the ligand conformations. At least 10 conformations were generated, and the model with the least binding energy and RMSD was chosen for further analysis. All generated docked structures were visualized using CCP4mg [28].

## 3. Results

### 3.1. Identification of the 43.8-kDa Protein from Shigella sonnei

**Figure 1:**
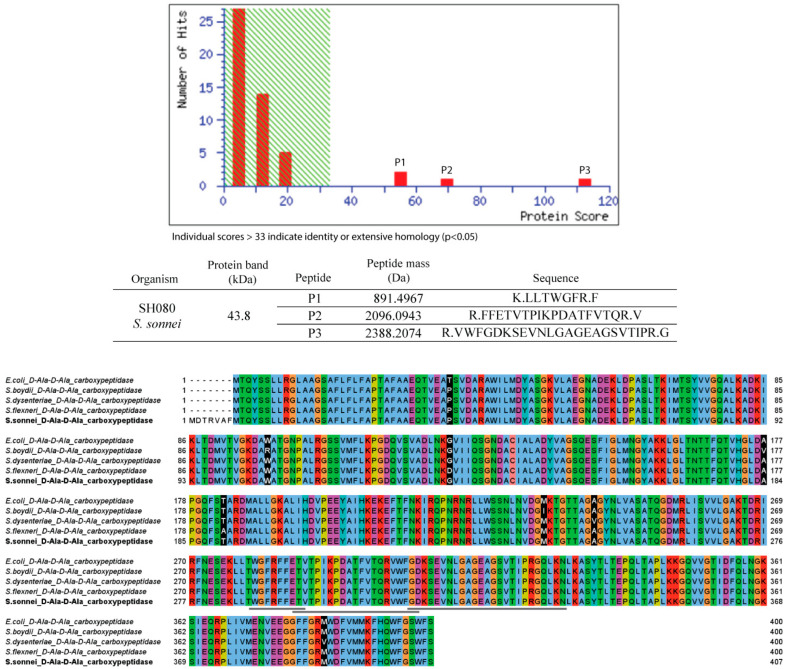
Mass spectrometry analysis and sequence alignment of D-Ala-D-Ala carboxypeptidase from *Shigella sonnei* and its orthologs. Mass spectrometry analysis was performed using MALDI-ToF technique, which generated three tryptic-digested peptides (891, 2096 and 2388 Da). Multiple sequence alignment was carried out using Clustal Omega on Jalview [22]. The protein sequence is highly conserved across the bacterial species. However, D-Ala-D-Ala carboxypeptidase from *S. sonnei* is 7 residues longer than D-Ala-D-Ala carboxypeptidases from *Escherichia coli*, *Shigella boydii*, *Shigella dysenteriae* and *Shigella flexneri*. The 7-residue sequence is probably a non-coding region. Variations across the bacterial species are highlighted as white on black. The three tryptic-digested peptides are underlined.

A previous study has shown that the 43.8-kDa outer membrane-associated protein from *S. sonnei* was able to uniquely recognize IgA and IgG from patients previously infected by the bacterium [21]. In order to identify and characterize the protein, mass spectrometry analysis using MALDI-ToF was carried out and the BLAST search against the non-redundant protein sequence database was performed to determine the protein type. Results from the BLAST search showed that the 43.8-kDa protein has high similarity with over 99% sequence identity and 100% query coverage with D-Ala-D-Ala carboxypeptidases (also known as PBP6) of *E. coli*, *S. flexneri*, *Shigella boydii* and *Shigella dysenteriae*. A multiple sequence alignment was carried out, comparing PBP6 from *S. sonnei* with its orthologs from other closely related bacterial species and the result showed that the protein is highly conserved, with a high degree of sequence homology (Figure 1). This is expected, as *S. sonnei* and other *Shigella* species are closely related and genetically similar to their ancestor *E. coli* [29]. 

### 3.2. Homology Model of D-Ala-D-Ala Carboxypeptidase (PBP6) from Shigella sonnei

Homology modelling builds 3D models of a protein using experimentally determined structures of related proteins as templates. Albeit its importance in the peptidoglycan biosynthesis, the structure of PBP6 from *S. sonnei* has not yet been determined. Due to its high sequence similarity with other PBP6s from the Enterobacteriaceae bacterial family, homology modelling was employed to construct the 3D structure of PBP6 from *S. sonnei* in this study. Thus, a BLAST search was performed against the PDB database to search for suitable templates and nine PBP5/6s from *E. coli*, *Vibrio cholerae*, *Haemophilus influenzae* and *Pseudomonas aeruginosa* (Table 1) were selected as templates to model the structure for the *S. sonnei* PBP6. Results from the BLAST search showed that the *S. sonnei* PBP6 has high similarity with 100% sequence identity and more than 84% query coverage with the selected templates. We modelled the *S. sonnei* PBP6, omitting the first 37 residues and the last 22 residues. This is a common strategy used for PBP5/6 expression and crystallization, because it removes the N-terminal signal peptide and the C-terminal hydrophobic portion of the protein [12]. 

**Figure 2:**
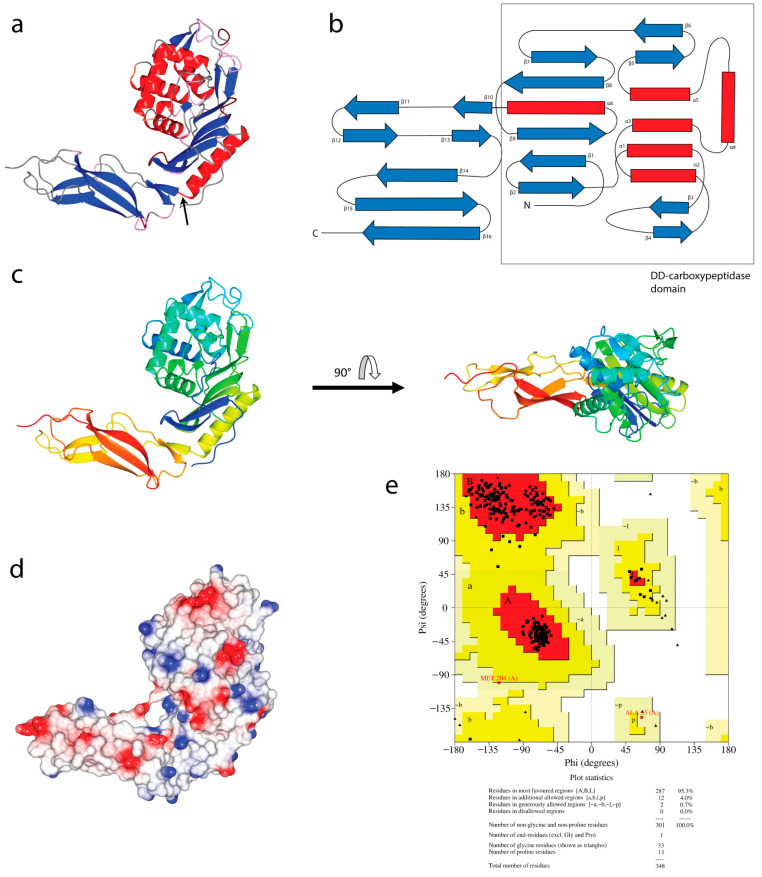
Overall 3D structure of PBP6 from *S. sonnei*. (**a**) The *S. sonnei* PBP6 adopts a usual PBP 5/6-like structure, which consists of two distinctive domains, a large N-terminal DD-carboxypeptidase domain and a smaller β-sheet rich C-terminal domain of unknown function (DUF). The arrow indicates the short linker that connects the two domains. (**b**) The topology diagram of the *S. sonnei* PBP6. The two domains are linked through a short linker between the last α-helix (α6) of the DD-carboxypeptidase domain and the first β-strand (β10) of the DUF. (**c**) Two orthogonal ribbon plot views of the *S. sonnei* PBP6, colored on a gradient from the N (blue) to the C (red) terminus. (**d**) An electrostatic surface plot of the *S. sonnei* PBP6. (**e**) The Ramachandran plot of the protein 3D model.

Figure 2 shows the overall structure of PBP6 from *S. sonnei*; it forms two distinctive domains, a large N-terminal DD-carboxypeptidase domain and a smaller β-sheet rich C-terminal domain of unknown function (DUF). The two main domains are linked through a short linker between the last α-helix of the DD-carboxypeptidase domain and the first β-strand of the DUF (Figure 2b). As in PBP6 from *E. coli*, the overall structure of the *S. sonnei* PBP6 consists of six α-helices and sixteen β-strands, in which the topology of the protein resembles those of class A β-lactamases [12]. The DD-carboxypeptidase domain is formed from five α-helices packed against a pseudopilin-like fold (a single α-helix packed against five antiparallel β-strands) [30], with extensive loop regions that form the active site of the protein. Evaluation of the stereochemical quality of the model by Ramachandran plot using PROCHECK [26] indicated that >95.3% (287 residues) of the residues have psi and phi angles in the most favored regions, with no residues in the disallowed region (Figure 2e).

**Figure 3:**
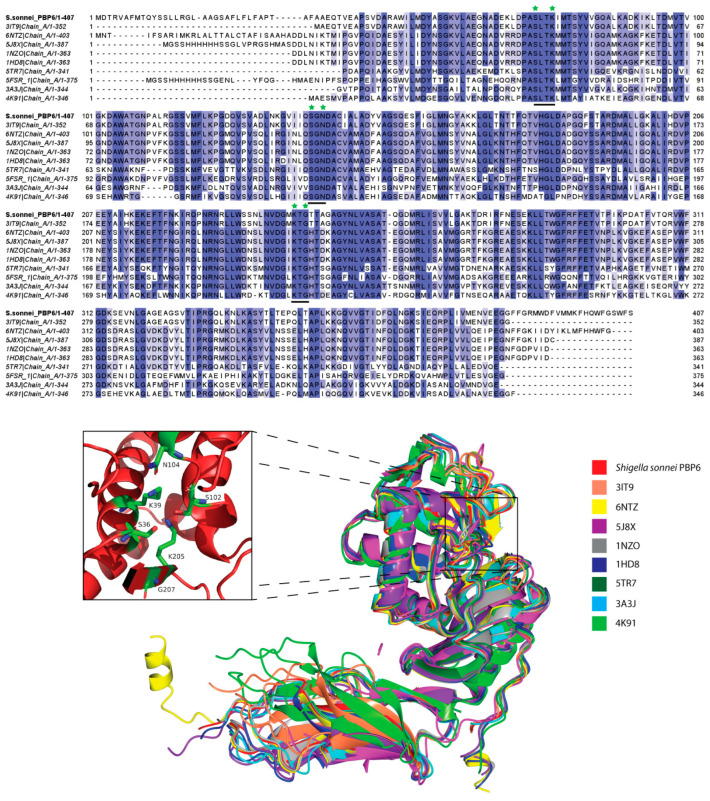
Sequence alignment and superimpositions of PBP6 from *S. sonnei* and the templates used in the protein modelling. Multiple sequence alignment using Clustal Omega on Jalview [22], which showed a high degree of sequence similarity among the templates. The three sequence motifs S-L-T-K, S-G-N and K-T-G (underlined) are conserved in all proteins and the catalytic active binding sites S36, K39, S102, N104, K205 and G207, located in a groove (bottom panel) within the DD-carboxypeptidase domain, are labelled with a star (top panel).

As expected from its sequence similarity, the overall structure of the *S. sonnei* PBP6 resembles that of PBP6 from *E. coli* [12], with a root-mean-square deviation (RMSD) of 0.3 Å between the Cα traces of two proteins (981 atoms aligned) (Figure 3). The loop (residues 67–91) that formed the active site groove of the *E. coli* PBP6 is bent slightly inwards, compared to the similar loop in the *S. sonnei* PBP6. The loop is important in the activity of the carboxypeptidase, as it makes extensive contacts with the S-*X*-N motif, essential in the deacylation of the acyl–enzyme complex [13]. Multiple sequence alignment suggested that PBP 5/6 templates used in the protein homology modelling share a high degree of sequence similarity. Like other PBPs, they also contain unique conserved motifs S-*X*-*X*-K, S-*X*-N and K-T-G, forming the active sites that are essential for the substrate catalysis [8,11]. These motifs also can be found in serine-based β-lactamases, such as AmpC and TEM-1 [12]. In the case of the *S. sonnei* PBP6, the three sequence motifs are S-L-T-K, S-G-N and K-T-G, where the first serine (S36) is the catalytic nucleophile and is located within the loop between β2 and α1 of the DD-carboxypeptidase domain (Figure 3). In PBP2, S310 of the S-X-X-K motif is the site of acylation by both the peptide substrate and β-lactams, and is important for the substrate binding and catalysis, where mutation at the position 310 to alanine has been shown to completely abolish the binding with ceftriaxone [31].

### 3.3. Molecular Docking of PBP6 from Shigella sonnei with Its Pentapeptide Substrate and β-Lactam Antibiotic Inhibitors

Crystal structures of PBP6 from *E. coli* have been previously reported [12]. The study describes the structural basis of interactions between PBP6 and its peptidoglycan substrate fragment containing the full pentapeptide in a pre-acylation complex; this is the first for a PBP as well as with its antibiotic ampicillin inhibitor, which provide valuable information on the molecular interactions vital for ligand binding and catalysis by DD-carboxypeptidases in general. Most importantly, the protein–substrate complex structure provides a template for models of cell wall biosynthesis by PBPs and in addition, the protein–inhibitor complex structure presents substantial evidence for the molecular recognition by β-lactam antibiotics for PBPs [12].

**Figure 4:**
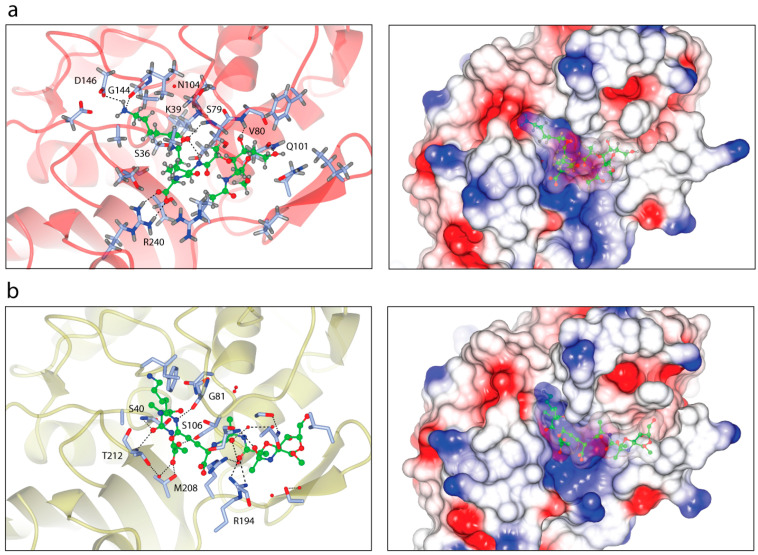
PBP6 complex structures with the pentapeptide substrate. (**a**) Interfacial contacts between the *S. sonnei* PBP6 (red) with the pentapeptide substrate (green). The substrate made hydrogen bonding interactions with residues S79, V80, Q101, G144, D146 and R240 of the *S. sonnei* PBP6. The electrostatic surface of the complex is in the right panel. (**b**) Interfacial contacts between the *E. coli* PBP6 (PDB: 3ITB) with the pentapeptide substrate [12]. Ribbon view of the substrate (green) in the active site groove of the *E. coli* PBP6 (gold), which made contacts with residues S40, G81, R194, T212 and M208. R194 establishes both a salt bridge and a water-mediated hydrogen bond with the pentapeptide. The electrostatic surface of the complex is in the right panel.

In order to demonstrate the intermolecular interaction between PBP6 from *S. sonnei* and its peptidoglycan substrate, we established *in silico* binding analysis and molecular docking between the two molecules, based on the previously reported structures of *E. coli* PBP6 [12,23]. The full pentapeptide used in this study was extracted from the crystal structure of *E. coli* PBP6 (PDB: 3ITB) [12] and a docking simulation was performed at the active sites of PBP6. The structure revealed that the pentapeptide was aligned in the active site groove of the DD-carboxypeptidase domain, in a similar fashion to that of the *E. coli* PBP6 (Figure 4). The docked complex of *S. sonnei* PBP6 with pentapeptides showed that the substrate bound to the active site groove via hydrogen bonding interactions with the residues S79, V80, Q101, G144, D146 and R240 (Figure 4a). Although the substrate was not in direct contact with the catalytic active sites of PBP6, the functionally important residues of PBP6 that formed the active site groove interacted with the substrate. These flexible loops may undergo conformational changes upon binding, which may bring the pentapeptide closer to the catalytic active sites of PBP6, particularly the catalytic nucleophile S36 that lies within the loop between β2 and α1 of the DD-carboxypeptidase domain and is in close proximity to the substrate. This may subsequently activate the acylation of the pentapeptide, ultimately leading to the transpeptidation of peptidoglycan biosynthesis. However, as for the *E. coli* PBP6, S40 has been shown to form a hydrogen bond with the D-Ala residue of the pentapeptide (Figure 4b) [12].

**Figure 5:**
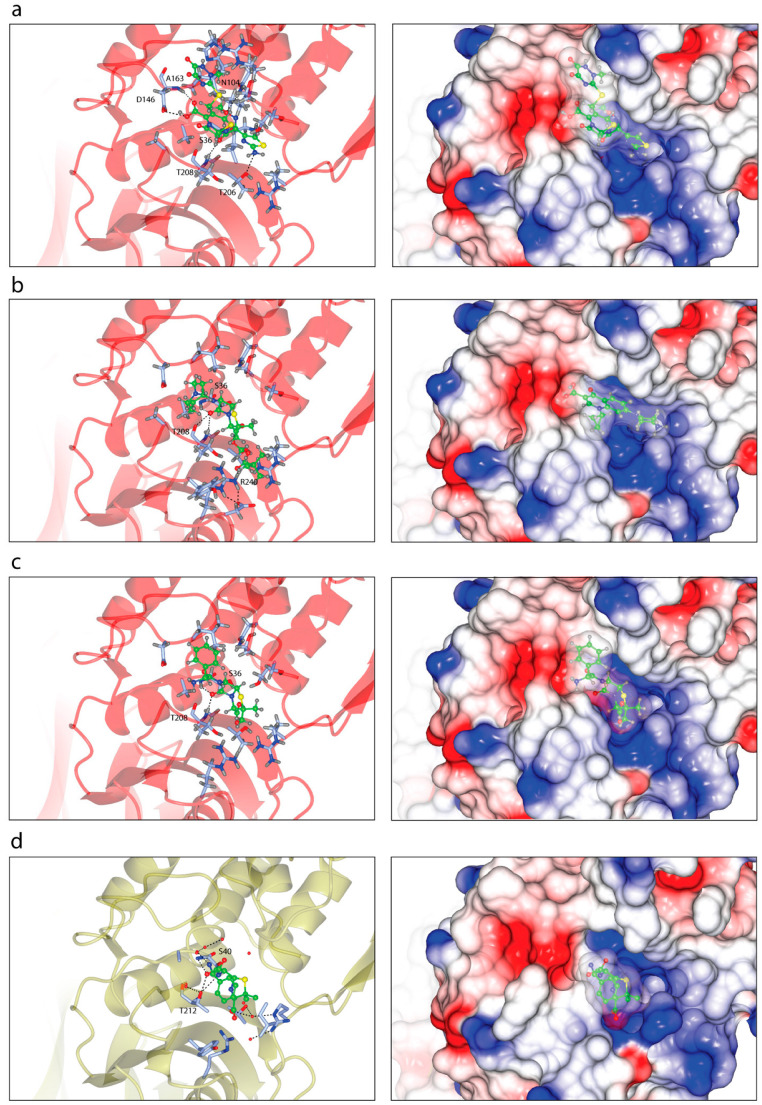
PBP6 complex structures with the β-lactam antibiotics. (**a**) Interfacial contacts between the *S. sonnei* PBP6 (red) with ceftriaxone (green). The antibiotic made five hydrogen bonding interactions with the residues N104, D146, A163, T206 and T208 of the *S. sonnei* PBP6. The electrostatic surface of the complex is in the right panel. (**b**) Interfacial contacts between the *S. sonnei* PBP6 (red) with pivmecillinam (green). There are four hydrogen bonds formed between pivmecillinam and the residues S36, T208 and R240 of the *S. sonnei* PBP6. (**c**) Interfacial contacts between the *S. sonnei* PBP6 (red) with ampicillin (green). There are two hydrogen bonding interactions formed between ampicillin and the residues S36 and T208. (**d**) Interfacial contacts between the *E. coli* PBP6 (gold) with ampicillin (green) in the acyl–enzyme complex (PDB: 3ITA) [12].

Given the importance of the β-lactam antibiotics, such as pivmecillinam and ceftriaxone, in the treatment of shigellosis, as recommended by the WHO as the second-line treatment in all age groups [6], we sought to evaluate the molecular interactions between PBP6 and these β-lactam antibiotics. The structures of pivmecillinam and ceftriaxone, retrieved from PubChem, were used in the protein-ligand analysis, performed using AutoDock Vina, as implemented in Chimera [27]. From the molecular docking simulations, we found that both β-lactam antibiotics can fit into the active site groove of the DD-carboxypeptidase domain, although in slightly different orientations, possibly owing to their different structures (Figure 5). The docked complex of PBP6 with ceftriaxone, a third-generation cephalosporin, showed that the β-lactam inhibitor bound to the groove via hydrogen bonding interactions with the residues N104, D146, A163, T206 and T208; completely shielded the catalytic nucleophile S36 from access by the substrate (Figure 5a). Moreover, the cephem group of ceftriaxone was oriented in close proximity to S36, which is important for nucleophilic attack of the β-lactam carbonyl by the serine residue and may eventually may result in the opening of the β-lactam ring and formation of a stable and long-lived acylated complex [8,10]. Furthermore, the active site N104 made contact with the methoxyimino group of ceftriaxone via a hydrogen bond and additionally, T206 and T208 that flanked the active site G207 formed hydrogen bonds with the amino and methoxyimino groups of ceftriaxone, respectively (Figure 5a).

However, unlike ceftriaxone—which was predicted to shield the catalytic site but did not interact the catalytic nucleophile S36—pivmecillinam had direct contact with S36 via the carbonyl group of its penam (Figure 5b). This is an important finding, as it shows a direct interaction between the inhibitor and the catalytic nucleophile S36, which is the key residue in the substrate catalysis. The serine hydrogen atom is involved in hydrogen bonding contact (2.4 Å) with the carbonyl oxygen atom of the penam ring, whereas the serine Oγ atom is involved in van der Waals contact (4.8 Å) with the carbonyl carbon atom of the penam ring, poised with proper orientation for the nucleophilic attack (Figure 5b). Compared to ceftriaxone, which formed five hydrogen bonds, pivmecillinam established four hydrogen bonds with PBP6. The other three were with T208 and R204, and these established strong hydrogen bonding interactions, predicted at 2.1/2.9 and 2.0 Å, respectively.

Similarly, the docked complex of PBP6 with ampicillin showed that the serine hydrogen atom is involved in hydrogen bonding contact (2.4 Å) with the carbonyl oxygen atom of the penam ring of ampicillin, but compared to pivmecillinam, ampicillin had only two hydrogen bonding contacts with PBP6. The other contact was a strong 1.8-Å hydrogen bond with T208 (Figure 5c). Albeit more contacts between the two molecules, ampicillin had fewer hydrogen bonds compared to pivmecillinam, which may explain the increasing antimicrobial resistance reported for the antibiotic [6]. As in the case of the *E. coli* PBP6, S40 has been shown to form a hydrogen bond with the penam ring of ampicillin in the acyl–enzyme complex, but relative to the pre-acylation complex with the pentapeptide substrate (Figure 4b), there are fewer contacts formed between PBP6 and ampicillin [12]. Additionally, the side chain of T212 is also involved in a hydrogen bond with the amide group of ampicillin (Figure 5d).

## 4. Discussion

Elucidating the complex structures between the *S. sonnei* PBP6 and its substrates would allow us to understand the molecular interactions and conformational changes, important for ligand recognition and catalysis by the enzyme. Together with the previous studies on other D-Ala-D-Ala carboxypeptidases, such as the *E. coli* PBP5 [14] and the *E. coli* PBP6 [12], they provide insights on how β-lactam antibiotics, such as ceftriaxone and pivmecillinam, inhibit PBPs by mimicking the peptidoglycan substrate. Our in depth understanding of these biological mechanisms at the molecular level will be helpful to guide us in the development of effective drugs to treat *S. sonnei* infections. *S. sonnei* has been continuously becoming resistant to antibiotics and shows an extraordinary ability to acquire antimicrobial resistance genes from other pathogenic bacterial species, such as *Klebsiella* spp. and *E. coli* [3,4]. In this particular context, various studies have analyzed the antimicrobial phenotypes and molecular mechanisms underlying resistance in *Shigella* spp. For instance, a previous study has focused on genetic characterization and antimicrobial resistance of *Shigella* spp., where ESBL genes, such as *bla*_TEM-1_, *bla*_CTX-M_, *bla*_OXA-1_, and *bla*_SHV-12_, are found to be dominant in the *S. sonnei* isolates. Resistance to ampicillin is the most common, in which 97.7% of the total 474 *Shigella* isolates are resistant to the broad-spectrum antibiotic, whereas 26.0% of the isolates are resistant to ceftriaxone, the third-generation cephalosporin [32]. Another study has reported that a resistance rate of 95.1% to ampicillin is observed among *Shigella* isolates, with 53.7% of the isolates carrying ESBL genes [33]. This is worrying, as these studies indicate an alarming increase in the ESBL production of *Shigella* spp. that confer the resistance to β-lactam antibiotics, such as pivmecillinam and ceftriaxone, which are currently used as the second-line treatment against shigellosis [5]. Even though PBPs are important targets for the β-lactam antibiotics, the molecular interactions between the *S. sonnei* PBP6 and its substrate, as well as antibiotic inhibitors, have not been structurally evaluated. As the high-resolution 1.80-Å crystal structure of the *E. coli* PBP6 in complex with the pentapeptide substrate has been published [12], molecular interaction observations of the *S. sonnei* PBP6 are warranted. *Shigella* spp. and *E. coli* are closely related and genetically similar species [29]. For example, the *S. sonnei* PBP6 shares 99% sequence identity with PBP6 from *E. coli* (Figure 1). Thus, using protein homology modelling, we modelled the structure of PBP6 from *S. sonnei* and comprehensively examined the molecular interactions between PBP6 and its substrate and inhibitors.

D-Ala-D-Ala carboxypeptidases, as is the case with PBP6, cleave the peptide bond between the two terminal D-alanines of the pentapeptide stem during the cross-linking of the peptidoglycan polymers, creating a mesh-like structure [8]. Like other PBPs, PBP6 also contains active site sequence motifs that are vital for catalysis, including S-*X*-*X*-K, S-*X*-N and K-T-G. The S-*X*-*X*-K motif is the site of acylation by both the peptide substrate and β-lactams, and is important for the substrate binding and catalysis [8,11]. The acylation involves the nucleophilic attack by serine at the carbonyl carbon of the penultimate D-Ala residue in the pentapeptide substrate. Here, we showed that the pentapeptide substrate, despite the fact that it did not make direct contact with the catalytic active sites of PBP6, was positioned along the active site groove so that the functionally important residues that formed the active site interacted with the substrate. Most importantly, the catalytic nucleophile S36 that lies within the flexible loop between β2 and α1 of the DD-carboxypeptidase domain is in close proximity to the substrate (Figure 4a). This flexible loop may undergo conformational changes upon substrate binding and bring the pentapeptide closer to the catalytic active sites of PBP6. The serine residue is important in the acylation of PBPs, where mutation of S310 to alanine in PBP2 has been shown to completely abolish the binding with ceftriaxone [31]. Structural conformational changes in the loop within the active site groove play an important key element in the substrate binding and acylation. Mutations in the β3–β4 loop of PBP2 have been shown to destabilize the high-affinity state containing the inward conformation of the loop that is required for contact with ceftriaxone in the active site, thereby conferring resistance to the antibiotic by a low-affinity drug-binding state [34]. Mutations also prevent bending of the β3–β4 loop and hinder the rotation of the β3 strand essential to form the oxyanion hole for acylation, thus trapping ceftriaxone in a noncanonical configuration [31].

It is widely accepted that nucleophilic attack of the β-lactam carbonyl by the catalytic residue serine of transpeptidases causes the opening of the β-lactam ring and the formation of a stable and irreversible acyl–enzyme complex [8,10]. However, a previous study has shown that the acylation of the L,D-transpeptidase by the β-lactam nitrocefin is reversible, which may lead to its limited antimicrobial activity [35]. Although L,D-transpeptidases are structurally unrelated to D,D-transpeptidase PBPs and cleave L-Lys-D-Ala of the tetrapeptide stem [36], they also interact with the β-lactam antibiotics and are acylated by β-lactams, which may provide insights into its resistance mechanism in general. PBPs recognize β-lactams as they mimic the acyl-D-Ala-D-Ala portion of the peptidoglycan substrate. Together with the *E. coli* PBP6 [12], our *S. sonnei* PBP6 complexes with the pentapeptide substrate, as well as the two β-lactam antibiotics, provide structural context to understand this mimicry and additionally give insights into the inhibition mechanism of β-lactams. Our structures (Figure 5) revealed that the functional β-lactam rings fit in closely identical positions compared to the pentapeptide substrate (Figure 4a). The β-lactam rings of all β-lactams studied here formed hydrogen bonds with T208, proving the importance of this residue in ligand recognition and binding. Furthermore, pivmecillinam made a hydrogen bond with R240, as did the substrate stem peptide. Although ceftriaxone did not make any direct contacts with the catalytic nucleophile S36, unlike pivmecillinam and ampicillin did, all of them were positioned in close proximity to the catalytic residue, allowing PBP6 to recognize and react with the β-lactams, forming an acyl–enzyme complex in the same way as it did with the pentapeptide substrate. 

## 5. Conclusions

Elucidating the complex structures between the *S. sonnei* PBP6 and its ligands not only provides information on their intermolecular interactions but can also be utilized to develop high-affinity and specialized PBP6 inhibitors. Like other PBPs, PBP6 recognizes β-lactams as they mimic the D-Ala-D-Ala moiety. From our molecular docking simulations, we found that the pentapeptide substrate and the β-lactam antibiotics can fit into the active site groove of the DD-carboxypeptidase domain, in close proximity to the catalytic nucleophile S36 for the nucleophilic attack. Two residues, T208 and R240, were found to be important in ligand recognition, where they made strong hydrogen bonds with β-lactams and pentapeptides, respectively. Our current computational analysis provides valuable information on the molecular interactions essential for ligand recognition and catalysis by PBP6. Understanding these interactions will be helpful in the development of new effective drugs to treat *S. sonnei* infections. Further *in vitro* studies using the *S. sonnei* PBP6 are warranted to ensure the specificity of the intermolecular interactions between the protein and its ligands.

## Figures and Tables

**Table 1:** Templates used in the protein homology modelling and the BLAST search result against PDB database with the 43.8-kDa protein from *S. sonnei*.

PDB ID	Organism	Title	Sequence Coverage/Identity (%)	*E*-Value
3IT9	*Escherichia coli*	Chain A, crystal structure of PBP6 from *Escherichia coli* in apo state	85.0/100.0	0.0
6NTZ	*Escherichia coli*	Chain A, crystal structure of *Escherichia coli* PBP5-meropenem	88.0/64.4	4 × 10^−168^
5J8X	*Escherichia coli*	Chain A, crystal structure of *Escherichia coli* PBP5 with 2C	86.0/65.0	3 × 10^−161^
1NZO	*Escherichia coli*	Chain A, crystal structure of wild type PBP5 from *Escherichia coli*	84.0/65.2	2 × 10^−158^
1HD8	*Escherichia coli*	ChainA, crystal structure of a deacylation-defective mutant of PBP5 at 2.3 Å resolution	84.0/64.9	3 × 10^−157^
5TR7	*Vibrio cholerae*	Chain A, crystal structure of a putative D-alanyl-D-alanine carboxypeptidase from *Vibrio cholerae*	84.0/55.8	1 × 10^−128^
5FSR	*Escherichia coli*	Chain A, crystal structure PBP6b from *Escherichia coli*	84.0/49.6	2 × 10^−115^
3A3J	*Haemophilus influenzae*	Chain A, crystal structure of PBP5 from *Haemophilus influenzae*	84.0/53.8	1 × 10^−114^
4K91	*Pseudomonas aeruginosa*	Chain A, crystal structure of PBP5 from *Pseudomonas aeruginosa* in apo state	84.0/47.3	7 × 10^−104^

## Data Availability

All data relevant to this review are included in the text and references.

## References

[1] Gu B., Cao Y., Pan S., Zhuang L., Yu R., Peng Z., Qian H., Wei Y., Zhao L., Liu G. (2012). Comparison of the prevalence and changing resistance to nalidixic acid and ciprofloxacin of Shigella between Europe-America and Asia-Africa from 1998 to 2009. Int. J. Antimicrob. Agents.

[2] Thompson C.N., Duy P.T., Baker S. (2015). The Rising Dominance of Shigella sonnei: An Intercontinental Shift in the Etiology of Bacillary Dysentery. PLoS Negl. Trop. Dis..

[3] Rashid H., Rahman M. (2015). Possible transfer of plasmid mediated third generation cephalosporin resistance between Escherichia coli and Shigella sonnei in the human gut. Infect. Genet. Evol..

[4] Pai H., Choi E.H., Lee H.J., Hong J.Y., Jacoby G.A. (2001). Identification of CTX-M-14 extended-spectrum beta-lactamase in clinical isolates of Shigella sonnei, Escherichia coli, and Klebsiella pneumoniae in Korea. J. Clin. Microbiol..

[5] WHO (2005). Guidelines for the Control of Shigellosis, Including Epidemics due to Shigella Dysenteriae Type 1.

[6] Williams P.C.M., Berkley J.A. (2018). Guidelines for the treatment of dysentery (shigellosis): A systematic review of the evidence. Paediatr. Int. Child Health.

[7] Bush K., Bradford P.A. (2016). β-Lactams and β-Lactamase Inhibitors: An Overview. Cold Spring Harb. Perspect. Med..

[8] Sauvage E., Kerff F., Terrak M., Ayala J.A., Charlier P. (2008). The penicillin-binding proteins: Structure and role in peptidoglycan biosynthesis. FEMS Microbiol. Rev..

[9] Satta G., Cornaglia G., Mazzariol A., Golini G., Valisena S., Fontana R. (1995). Target for bacteriostatic and bactericidal activities of beta-lactam antibiotics against Escherichia coli resides in different penicillin-binding proteins. Antimicrob. Agents Chemother..

[10] Macheboeuf P., Contreras-Martel C., Job V., Dideberg O., Dessen A. (2006). Penicillin binding proteins: Key players in bacterial cell cycle and drug resistance processes. FEMS Microbiol. Rev..

[11] Scheffers D.-J., Pinho M.G. (2005). Bacterial Cell Wall Synthesis: New Insights from Localization Studies. Microbiol. Mol. Biol. Rev..

[12] Chen Y., Zhang W., Shi Q., Hesek D., Lee M., Mobashery S., Shoichet B.K. (2009). Crystal structures of penicillin-binding protein 6 from Escherichia coli. J. Am. Chem. Soc..

[13] Nicholas R.A., Krings S., Tomberg J., Nicola G., Davies C. (2003). Crystal structure of wild-type penicillin-binding protein 5 from *Escherichia coli*: Implications for deacylation of the acyl-enzyme complex. J. Biol. Chem..

[14] Caveney N.A., Caballero G., Voedts H., Niciforovic A., Worrall L.J., Vuckovic M., Fonvielle M., Hugonnet J.E., Arthur M., Strynadka N.C.J. (2019). Structural insight into YcbB-mediated beta-lactam resistance in Escherichia coli. Nat. Commun..

[15] Brem J., Cain R., Cahill S., McDonough M.A., Clifton I.J., Jiménez-Castellanos J.C., Avison M.B., Spencer J., Fishwick C.W.G., Schofield C.J. (2016). Structural basis of metallo-β-lactamase, serine-β-lactamase and penicillin-binding protein inhibition by cyclic boronates. Nat. Commun..

[16] Davies C., White S.W., Nicholas R.A. (2001). Crystal structure of a deacylation-defective mutant of penicillin-binding protein 5 at 2.3-A resolution. J. Biol. Chem..

[17] Kawai F., Clarke T.B., Roper D.I., Han G.J., Hwang K.Y., Unzai S., Obayashi E., Park S.Y., Tame J.R.H. (2010). Crystal structures of penicillin-binding proteins 4 and 5 from Haemophilus influenzae. J. Mol. Biol..

[18] Smith J.D., Kumarasiri M., Zhang W., Hesek D., Lee M., Toth M., Vakulenko S., Fisher J.F., Mobashery S., Chen Y. (2013). Structural analysis of the role of Pseudomonas aeruginosa penicillin-binding protein 5 in β-lactam resistance. Antimicrob. Agents Chemother..

[19] Zhang W., Shi Q., Meroueh S.O., Vakulenko S.B., Mobashery S. (2007). Catalytic mechanism of penicillin-binding protein 5 of Escherichia coli. Biochemistry.

[20] Shi Q., Meroueh S.O., Fisher J.F., Mobashery S. (2008). Investigation of the mechanism of the cell wall DD-carboxypeptidase reaction of penicillin-binding protein 5 of Escherichia coli by quantum mechanics/molecular mechanics calculations. J. Am. Chem. Soc..

[21] Harikrishnan H., Banga Singh K.K., Ismail A. (2017). Outer membrane proteins analysis of Shigella sonnei and evaluation of their antigenicity in Shigella infected individuals. PLoS ONE.

[22] Sievers F., Wilm A., Dineen D., Gibson T.J., Karplus K., Li W., Lopez R., McWilliam H., Remmert M., Söding J. (2011). Fast, scalable generation of high-quality protein multiple sequence alignments using Clustal Omega. Mol. Syst. Biol..

[23] Peters K., Kannan S., Rao V.A., Biboy J., Vollmer D., Erickson S.W., Lewis R.J., Young K.D., Vollmer W. (2016). The Redundancy of Peptidoglycan Carboxypeptidases Ensures Robust Cell Shape Maintenance in Escherichia coli. MBio.

[24] Kuntal B.K., Aparoy P., Reddanna P. (2010). EasyModeller: A graphical interface to MODELLER. BMC Res. Notes.

[25] Eisenberg D., Lüthy R., Bowie J.U. (1997). VERIFY3D: Assessment of protein models with three-dimensional profiles. Methods Enzymol..

[26] Laskowski R.A., Rullmann J.A.C., MacArthur M.W., Kaptein R., Thornton J.M. (1996). AQUA and PROCHECK-NMR: Programs for checking the quality of protein structures solved by NMR. J. Biomol. NMR.

[27] Trott O., Olson A.J. (2010). AutoDock Vina: Improving the speed and accuracy of docking with a new scoring function, efficient optimization, and multithreading. J. Comput. Chem..

[28] McNicholas S., Potterton E., Wilson K.S., Noble M.E.M. (2011). Presenting your structures: The CCP4mg molecular-graphics software. Acta Crystallogr. D Biol. Crystallogr..

[29] Chattaway M.A., Schaefer U., Tewolde R., Dallman T.J., Jenkins C. (2017). Identification of Escherichia coli and Shigella Species from Whole-Genome Sequences. J. Clin. Microbiol..

[30] Salleh M.Z., Karuppiah V., Snee M., Thistlethwaite A., Levy C.W., Knight D., Derrick J.P. (2019). Structure and Properties of a Natural Competence-Associated Pilin Suggest a Unique Pilus Tip-Associated DNA Receptor. MBio.

[31] Singh A., Turner J.M., Tomberg J., Fedarovich A., Unemo M., Nicholas R.A., Davies C. (2020). Mutations in penicillin-binding protein 2 from cephalosporin-resistant Neisseria gonorrhoeae hinder ceftriaxone acylation by restricting protein dynamics. J. Biol. Chem..

[32] Wang Y., Ma Q., Hao R., Zhang Q., Yao S., Han J., Ren B., Fan T., Chen L., Xu X. (2019). Antimicrobial resistance and genetic characterization of Shigella spp. in Shanxi Province, China, during 2006–2016. BMC Microbiol..

[33] Aminshahidi M., Arastehfar A., Pouladfar G., Arman E., Fani F. (2017). Diarrheagenic Escherichia coli and Shigella with High Rate of Extended-Spectrum Beta-Lactamase Production: Two Predominant Etiological Agents of Acute Diarrhea in Shiraz, Iran. Microb. Drug Resist..

[34] Fenton B.A., Tomberg J., Sciandra C.A., Nicholas R.A., Davies C., Zhou P. (2021). Mutations in PBP2 from ceftriaxone-resistant Neisseria gonorrhoeae alter the dynamics of the β3–β4 loop to favor a low-affinity drug-binding state. J. Biol. Chem..

[35] Edoo Z., Arthur M., Hugonnet J.E. (2017). Reversible inactivation of a peptidoglycan transpeptidase by a β-lactam antibiotic mediated by β-lactam-ring recyclization in the enzyme active site. Sci. Rep..

[36] Biarrotte-Sorin S., Hugonnet J.E., Delfosse V., Mainardi J.L., Gutmann L., Arthur M., Mayer C. (2006). Crystal structure of a novel beta-lactam-insensitive peptidoglycan transpeptidase. J. Mol. Biol..

